# *PIK3CA* mutations, phosphatase and tensin homolog, human epidermal growth factor receptor 2, and insulin-like growth factor 1 receptor and adjuvant tamoxifen resistance in postmenopausal breast cancer patients

**DOI:** 10.1186/bcr3606

**Published:** 2014-01-27

**Authors:** Karin Beelen, Mark Opdam, Tesa M Severson, Rutger HT Koornstra, Andrew D Vincent, Jelle Wesseling, Jettie J Muris, Els MJJ Berns, Jan B Vermorken, Paul J van Diest, Sabine C Linn

**Affiliations:** 1 Departments of Molecular Biology, The Netherlands Cancer Institute, Amsterdam, The Netherlands; 2 Departments of Biometrics, The Netherlands Cancer Institute, Amsterdam, The Netherlands; 3 Departments of Pathology, The Netherlands Cancer Institute, Amsterdam, The Netherlands; 4 Department of Medical Oncology, Erasmus University Medical Center- Cancer Center, Rotterdam, The Netherlands; 5 Department of Medical Oncology, Antwerp University Hospital, Edegem, Belgium; 6 Department of Pathology, University Medical Center Utrecht, Utrecht, The Netherlands; 7 Departments of Medical Oncology, The Netherlands Cancer Institute, Amsterdam, The Netherlands

## Abstract

**Introduction:**

Inhibitors of the phosphatidylinositol-3-kinase/protein kinase B/mammalian target of rapamycin (PI3K/AKT/mTOR) pathway can overcome endocrine resistance in estrogen receptor (ER) α-positive breast cancer, but companion diagnostics indicating PI3K/AKT/mTOR activation and consequently endocrine resistance are lacking. *PIK3CA* mutations frequently occur in ERα-positive breast cancer and result in PI3K/AKT/mTOR activation *in vitro*. Nevertheless, the prognostic and treatment-predictive value of these mutations in ERα-positive breast cancer is contradictive. We tested the clinical validity of *PIK3CA* mutations and other canonic pathway drivers to predict intrinsic resistance to adjuvant tamoxifen. In addition, we tested the association between these drivers and downstream activated proteins.

**Methods:**

Primary tumors from 563 ERα-positive postmenopausal patients, randomized between adjuvant tamoxifen (1 to 3 years) versus observation were recollected. *PIK3CA* hotspot mutations in exon 9 and exon 20 were assessed with Sequenom Mass Spectometry. Immunohistochemistry was performed for human epidermal growth factor receptor 2 (HER2), phosphatase and tensin homolog (PTEN), and insulin-like growth factor 1 receptor (IGF-1R). We tested the association between these molecular alterations and downstream activated proteins (like phospho-protein kinase B (p-AKT), phospho-mammalian target of rapamycin (p-mTOR), p-ERK1/2, and p-p70S6K). Recurrence-free interval improvement with tamoxifen versus control was assessed according to the presence or absence of canonic pathway drivers, by using Cox proportional hazard models, including a test for interaction.

**Results:**

*PIK3CA* mutations (both exon 9 and exon 20) were associated with low tumor grade. An enrichment of *PIK3CA* exon 20 mutations was observed in progesterone receptor- positive tumors. *PIK3CA* exon 20 mutations were not associated with downstream-activated proteins. No significant interaction between *PIK3CA* mutations or any of the other canonic pathway drivers and tamoxifen-treatment benefit was found.

**Conclusion:**

*PIK3CA* mutations do not have clinical validity to predict intrinsic resistance to adjuvant tamoxifen and may therefore be unsuitable as companion diagnostic for PI3K/AKT/mTOR inhibitors in ERα- positive, postmenopausal, early breast cancer patients.

## Introduction

Recently, inhibitors of the phosphatidylinositol-3-kinase (PI3K)/AKT/mammalian target of rapamycin (mTOR) pathway have been introduced into the clinic to overcome endocrine resistance [1,2]. However, companion diagnostics for these new targeted drugs are lacking. Many molecular alterations in this pathway, as well as in the mitogen-activated protein kinase (MAPK) pathway, leading to its constitutive activation, have been described. Canonic pathway drivers are mutations in the *PIK3CA* gene [3], loss of expression or genetic alteration in the tumor-suppressor gene PTEN [4], and overexpression of growth factor receptors like human epidermal growth factor receptor 2 (HER2) and insulin-like growth factor 1 receptor (IGF-1R) [5].

*PIK3CA* mutations occur in about 20% to 25% of invasive ductal breast cancers and in approximately 40% of invasive lobular breast cancers [6], with hotspots in exon 9 (helical domain) and exon 20 (kinase domain). These mutations have been shown to result in *in vitro* activation of the PI3K/AKT/mTOR pathway [3], leading to endocrine resistance [7]. Nevertheless, the prognostic and predictive value regarding endocrine resistance of these mutations in ERα-positive breast cancer remains unclear. An important limitation of many conflicting clinical studies [8-12] is the analysis of these mutations in consecutive series of endocrine-treated patients, which is unsuitable to discern prognosis from prediction [13]. Only one previous study [14] analyzed these mutations in the context of a clinical trial that randomized between adjuvant tamoxifen and control. In this study, *PIK3CA* mutations did not predict endocrine resistance, but were associated with a decreased risk for local recurrence. In neoadjuvant endocrine therapy trials, *PIK3CA* mutation status was not associated with treatment-induced Ki67 changes, a surrogate marker for recurrence-free survival [15], nor with pathologic response [16], whereas the kinase domain mutations were associated with improved overall survival. Several other studies have suggested a relatively favorable survival in patients with kinase domain-mutated breast cancers [8,17], in comparison with patients without such mutated tumors.

Several other known molecular alterations in the PI3K and or the MAPK pathway have been studied for their validity to predict endocrine resistance. Loss of PTEN, a negative regulator of the PI3K/AKT/mTOR pathway, frequently occurs in breast cancer [18], but did not have clinical validity as a single marker in a previous study [14]. The same holds true for HER2 [19], although the clinical validity of IGF-1R has not been analyzed in the context of a randomized clinical trial.

The aim of our study was to investigate the prognostic and treatment-predictive value of different molecular alterations in the PI3K and/or MAPK pathways in postmenopausal breast cancer patients randomized between adjuvant tamoxifen and no systemic treatment. In addition, we studied the association between these molecular alterations and downstream-activated proteins in the PI3K and/or MAPK pathways.

## Methods

### Patients and material

We recollected primary tumor-tissue blocks from stage I through III postmenopausal breast cancer patients who were randomized (2:1) between 1-year tamoxifen (30 mg per day) and no adjuvant therapy (IKA trial, 1982 to 1994) [20,21]. Study data were part of the Oxford meta-analysis [22]. After 1989, based on two interim analyses showing a significant improvement in recurrence-free survival in lymph node-positive patients, node-positive patients in this trial skipped the first randomization, and all received 1 year of tamoxifen. After 1 year, a second randomization was performed to receive another 2 years of tamoxifen or to stop further treatment. In total, 1,662 patients were included. None of these patients received adjuvant chemotherapy. The patient characteristics and clinical outcome of the original study group (1,662 patients) were presented elsewhere [21].

Sufficient tumor material was available for 739 patients, who did not differ in prognostic factors from the total group (see Additional file 1: Table S1). After revision of ERα status as assessed with immunohistochemistry (IHC), a total of 563 ERα-positive tumors were used for subsequent analysis. We used a cutoff of ≥ 10% of positive tumor cells for ERα positivity, because this is a common practice in The Netherlands and, in addition, this would avoid the potential inclusion of basal-like tumors [23] in our analysis. The original trial was approved by the central ethics committee of the Netherlands Cancer Institute, and informed consent was obtained from all study participants. For this retrospective translational study, no additional consent was required, according to Dutch legislation [24], because the use of archival pathology leftover material does not interfere with patient care. Tumor tissue was handled according to the Dutch code of conduct for dealing responsibly with human tissue in the context of health research [25].

### Immunohistochemistry

Tissue microarrays (TMAs) were constructed by using formalin-fixed paraffin-embedded (FFPE) tumor blocks. A total of three (0.6 mm) cores per tumor were embedded in the TMAs that were stained for ERα, progesterone receptor (PgR), and HER2. ERα and PgR were considered positive when ≥10% of invasive cells showed nuclear reactivity. HER2 was considered positive when membranous staining was DAKO score 3. In case of a DAKO score 2, chromogenic *in situ* hybridization was performed. For tumors without sufficient cores in the TMA, whole slides were cut and assessed for ERα (*n* = 60), PgR (*n* = 55), and HER2 (*n* = 36). Tumor grade was scored on a hematoxylin-eosin-stained slide by using the modified Bloom-Richardson score [26].

Antibodies used for immunohistochemistry are shown in Additional file 1: Table S2. Immunohistochemistry for IGF-1R and PTEN was performed by using the Ventana Benchmark Ultra system (protocols can be found at [27]). For IGF-1R, membranous intensity was scored from 0 to 3. For PTEN protein expression, cytoplasmic intensity was scored from 0 to 3. The specificity of both antibodies was tested on a previously described series of metastatic breast cancer patients [28] for which we had FFPE material embedded in a TMA, as well as Agilent 44 K mRNA expression data. Results are depicted in Additional file 1: Figures S1 to S2. Immunohistochemistry for downstream phosphorylated (p) proteins in the PI3K/AKT/mTOR pathway, like p-AKT, p-extracellular signal-regulated kinase (ERK)1/2, p-mTOR, and p-p70S6K was performed as previously described [29].

The interobserver variability analyzed by using the (weighted) Cohen kappa coefficient is depicted in Additional file 1: Table S3. For further analyses, we used the scores produced by the first observer (MO).

### DNA isolation and *PIK3CA* mutation analysis

DNA was isolated by using a standard DNA-isolation protocol, as described in Appendix 1.

*PIK3CA* mutation status was assessed by using Sequenom mass spectrometry–based genotyping technology for *PIK3CA* hotspot mutations in exon 9 (E542K and E545K) and exon 20 (H1047L and H1047R). PCR primers and extension primers for the various mutations are listed in Additional file 1: Table S4.

### Statistics

The association of *PIK3CA* mutations, PTEN, HER2, and IGF-1R protein expression with known clinicopathologic factors was tested by using Fisher Exact tests. The association with downstream-activated proteins in the PI3K and/or MAPK pathways was evaluated by using linear by linear tests. Recurrence-free interval was defined as the time from the date of first randomization until the occurrence of a local, regional, or distant recurrence or breast cancer-specific death. Because a secondary contralateral breast tumor cannot be inferred from the molecular makeup of the primary tumor, whereas the other types of events can, in relation to tamoxifen resistance of the primary tumor, this was not considered an event, and these patients were censored at the date of their contralateral breast cancer.

We hypothesized that the presence of a molecular alteration in the PI3K and/or MAPK pathway is associated with tamoxifen resistance. In our primary analysis, we tested the clinical validity of these molecular alterations as single markers, analyzed as binary factor. Covariate adjusted Cox proportional hazard regression analyses were performed, including an interaction variable. The following molecular alterations were tested: *PIK3CA* mutation status (exon 9 mutant versus exon 9 wild-type and exon 20 mutant versus exon 20 wild-type imputed as separate factors in one model), HER2 (positive versus negative), PTEN (negative versus positive), and IGF-1R (score 3 versus score 0 to 2). In addition, we tested the interaction with tamoxifen for a composed variable, defined as any of these molecular alterations present versus no molecular alteration. Covariates included age (65 years or older versus younger than 65), grade (grade 3 versus grades 1 to 2), tumor size (T3 to T4 versus T1 to T2), HER2 status (positive versus negative), and PgR status (positive versus negative). All survival analyses were stratified for nodal status. Because of multiple co-primary end points, the level of significance was set at 0.01.

To assess the prognostic value of these molecular alterations, we analyzed their putative prognostic potential by using covariate adjusted Cox proportional-hazard regression analyses in the subgroup of patients who were randomized to the control arm. We did not use all patients and corrected for tamoxifen treatment because this correction would assume that all ERα-positive breast cancer patients would derive similar benefit from tamoxifen. Because the molecular alteration might be associated with tamoxifen resistance, simply correcting for the assumed tamoxifen benefit without a correction for a potential interaction between treatment and molecular alteration could bias the analysis for prognostic potential.

This study complied with reporting recommendations for tumor-marker prognostic studies (REMARK) criteria [30], as outlined in Additional file 1: Table S5.

## Results

### Associations between molecular alterations in PI3K/AKT/mTOR pathway and known prognostic factors and downstream-activated proteins

Genotyping for *PIK3CA* exon 9 mutations was successful in 488 ERα-positive tumor samples. Exon 20 mutations could be assessed in 491 tumor samples (Additional file 1: Figure S3). In total, 76 tumors harbored a *PIK3CA* exon 9 mutation (15.6%). Mutations in exon 20 were found in 89 (18.1%) of the tumors. In total, four tumors exhibited both exon 9 and exon 20 mutations. Overexpression/amplification of HER2 was seen in 41 (7.7%) of 530 tumors. The frequency of the different hot-spot mutations and the distribution of the intensity of IGF-1R protein expression are shown in Additional file 1: Table S6. PTEN protein expression could be assessed in 436 tumors, of which 82 (18.8%) did not show expression of PTEN. When PIK3CA exon 9 and exon 20 were compared with PIK3CA wild-type tumors, mutants were more often low grade. PIK3CA exon 9 mutations were associated with negative HER2 status, and for PIK3CA exon 20 mutations, an association with positive progesterone receptor status was observed. HER2-positive tumors were associated with positive lymph node status, high grade, and negative PgR status (Tables 1 and 2). In addition, PTEN-negative tumors were associated with negative PgR status.

**Table 1:** **Associations between *PIK3CA *** **mutation status and clinico-pathologic variables**

		***PIK3CA *** **mutation status**				
		**Wild-type**	**Exon 9 mutant**^**a**^		**Exon 20 mutant**^**a**^	
		***n *** **(%)**	***n *** **(%)**	***P *** **value**^**b**^	***n *** **(%)**	**P value **^**b**^
**Age**	**<65**	166 (51)	30 (39)	0.10	39 (44)	0.28
	**≥65**	161 (49)	46 (61)		50 (56)	
**Lymph node status**	**Negative**	177 (54)	39 (51)	0.70	55 (62)	0.23
	**Positive**	150 (46)	37 (49)		34 (38)	
**T stage**	**T1-2**	293 (90)	69 (91)	1.00	79 (89)	0.85
	**T3-4**	34 (10)	7 (9)		10 (11)	
**Grade**	**Grade 1-2**	198 (61)	59 (78)	0.005	65 (73)	0.04
	**Grade 3**	129 (39)	17 (22)		24 (27)	
**Progesterone receptor**	**Negative**	164 (50)	35 (46)	0.70	34 (38)	0.05
	**Positive**	159 (49)	38 (50)		54 (61)	
	**Missing**	4 (1)	3 (4)		1 (1)	
**HER2**	**Negative**	282 (86)	71 (93)	0.03	80 (90)	0.39
	**Positive**	29 (9)	1 (1)		5 (6)	
	**Missing**	16 (5)	4 (5)		4 (4)	
**Histologic subtype**	**Ductal**	236 (72)	56 (73)	1.00^c^	63 (71)	0.84^c^
	**Lobular**	36 (11)	8 (11)		8 (9)	
	**Other**	55 (17)	12 (16)		18 (20)	

^a^A total of four tumors exhibited both exon 9 and exon 20 mutations. ^b^Fisher Exact test for mutant versus wild type; only cases without missing values were analyzed. ^c^Fisher Exact test for ductal versus lobular.

**Table 2:** Associations between HER2, PTEN, IGF-1R, and clinico-pathologic variables

		**HER2 status**			**PTEN**			**IGF-1-R**		
		**Negative**	**Positive**		**Negative**	**Positive**		**Low (0–2)**	**High (3)**	
		***n *** **(%)**	***n *** **(%)**	***P*** **value**	***n *** **(%)**	***n *** **(%)**	***P *** **value**	***n *** **(%)**	***n *** **(%)**	***P*-value**
**Age**	**<65**	233 (48)	18 (44)	0.75	39 (48)	164 (46)	0.90	190 (47)	19 (49)	1.00
	**≥65**	256 (52)	23 (56)		43 (52)	190 (54)		210 (53)	20 (51)	
**Lymph node status**	**Negative**	276 (56)	16 (39)	0.03	42 (51)	189 (53)	0.81	211 (53)	22 (56)	0.74
	**Positive**	213 (44)	25 (61)		40 (49)	165 (47)		189 (47)	17 (44)	
**T stage**	**T1-2**	437 (89)	34 (83)	0.20	70 (85)	317 (89)	0.33	354 (89)	31 (80)	0.12
	**T3-4**	52 (11)	7 (17)		12 (15)	37 (11)		46 (11)	8 (20)	
**Grade**	**Grade 1-2**	342 (70)	9 (22)	<0.001	45 (55)	226 (64)	0.16	250 (63)	26 (67)	0.73
	**Grade 3**	147 (30)	32 (78)		37 (45)	128 (36)		150 (37)	13 (33)	
**Progesterone receptor**^**a**^	**Negative**	222 (45)	30 (73)	0.001	49 (60)	163 (46)	0.03	194 (49)	18 (46)	1.00
	**Positive**	261 (53)	11 (27)		32 (39)	190 (54)		205 (51)	20 (51)	
	**Missing**	6 (1)	0 (0)		1 (1)	1 (0)		1 (0)	1 (3)	
**HER2**^**a**^	**Negative**	489 (100)	0 (0)	na	77 (94)	313 (88)	0.08	357 (89)	36 (92)	0.56
	**Positive**	0 (0)	41 (100)		3 (4)	36 (10)		36 (9)	2 (5)	
	**Missing**	na	na		2 (2)	5 (1)		7 (2)	1 (3)	

^**a**^Only cases without missing values were analyzed.

**Figure 1:**
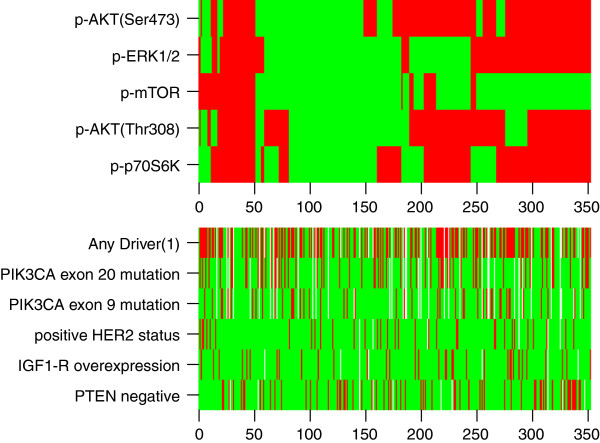
**Hierarchic clustering of the downstream-activated PI3K and/or MAPK proteins.** Heat map representing unsupervised hierarchic clustering of tumor samples and corresponding downstream activated proteins in the PI3K and/or MAPK pathways from patients for whom the status of all five proteins was known (*N* = 350). Patients are represented horizontally. Phosphorylated proteins are indicated vertically. Red represents high/any expression of phosphorylated protein, and green represents no/low expression of phosphorylated protein (dichotomization was performed according to the Akaike information criteria [29]). In addition, the presence (red) or absence (green) of different molecular alterations in the PI3K and or MAPK pathways is shown. ^(1)^Defined as the presence of either a *PIK3CA* mutation, positive HER2 status, or IGF-1R overexpression (IHC score 3).

We did not find significant associations between either *PIK3CA* exon 20 mutations or HER2 status and downstream-activated proteins in the PI3K pathway (Additional file 1: Table S7). *PIK3CA* exon 20 mutations were associated with higher p-ERK1/2 levels. Tumors with a *PIK3CA* exon 9 mutation were associated with higher p-AKT(Thr308) and p-ERK1/2 expression, but not with p-mTOR or p-p70S6K. Tumors that were scored as PTEN-negative had significantly lower levels of all the downstream-activated proteins than tumors that did express PTEN. Higher IGF-1R protein expression correlated with higher p-AKT (Ser 473) and p-p70S6K expression. Hierarchic clustering of the different downstream-activated proteins in the PI3K and/or MAPK pathway is shown in Figure 1. No clear enrichment appeared for any of the molecular alterations in tumors that express downstream activated proteins in the PI3K and/or MAPK pathway.

### PIK3CA mutations, loss of PTEN, and overexpression/amplification of HER2 and/or IGF-1R do not predict resistance to tamoxifen

**Figure 2:**
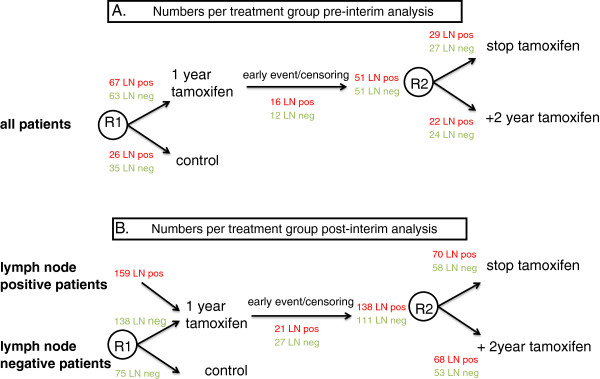
**Numbers of patients per randomization group before and after interim analysis.** Numbers of patients per randomization group before interim analysis **(A)** and after interim analysis **(B)**, for the total subset of 563 ERα-positive patients. From 1989, based on two interim analyses showing a significant improvement in recurrence-free survival among lymph node-positive patients, these node-positive patients were all allocated to the tamoxifen treatment arm (that is, skipped the first randomization). Numbers of lymph node-negative patients are depicted in green. In red are depicted the numbers of lymph node-positive patients. LN neg, lymph node negative; LN pos, lymph node positive; R1, randomization 1; R2, randomization 2.

Median follow-up of patients without a recurrence event is 7.8 years. The total number of events in the group of ERα-positive patients (*N* = 563) is 132. The number of patients in each treatment arm before and after interim analysis is shown in Figure 2. When stratified by nodal status, the hazard ratio (HR) for tamoxifen versus control in this cohort was 0.54 (95% confidence interval (CI), 0.36 to 0.83; *P* = 0.004). Known prognostic factors were equally divided over the treatment arms for all *PIK3CA* genotypes, with the exception of lymph node status, which can be explained by the change in randomization (Additional file 1: Table S8). In our primary analysis, patients with a tumor with either a *PIK3CA* exon 9 or exon 20 mutation did not derive significant benefit from tamoxifen (HR, 0.82 (95% CI, 0.22 to 3.04) and 0.77 (95% CI, 0.25 to 2.36), respectively (Table 3 and Additional file 1: Table S9) However, the interaction between *PIK3CA* mutations and tamoxifen was not significant. In addition, we did not observe a significant interaction between any of the other molecular alterations and tamoxifen, indicating that the presence or absence of these alterations by itself was not associated with a significant difference in tamoxifen efficacy in our series (Table 3). In addition, the composed variable indicating the presence of any of these molecular alterations did not show a significant interaction with tamoxifen (Table 3).

**Table 3:** Adjusted hazard ratios and interaction tests between PI3K and/or MAPK molecular alterations and tamoxifen

		**Number**	**adjusted HR**^**c **^**for tamoxifen versus control (95% confidence interval)**	**Adjusted *P *** **value for interaction**
***PIK3CA***	**Wild type**	308	0.51 (0.30-0.88)	
	**Exon 20 mutant**^a^	82	0.77 (0.25-2.36)	0.51
	**Exon 9 mutant**^a^	71	0.82 (0.22-3.04)	0.51
**PTEN**	**Negative**	78	0.44 (0.15-1.28)	0.63
	**Positive**	348	0.58 (0.34-0.98)	
**HER2**	**Negative**	479	0.52 (0.33-0.80)	0.52
	**Positive**	41	0.85 (0.19-3.95)	
**IGF-1R**	**Low (0–2)**	390	0.55 (0.33-0.91)	0.90
	**High (3)**	38	0.60 (0.20-1.76)	
**Any molecular alteration**	**Absent**	206	0.48 (0.22-1.02)	0.36
	**Present**^b^	151	0.76 (0.38-1.53)	

^a^Four tumors exhibited both exon 9 and exon 20 mutations. ^b^ Defined as the presence of either a *PIK3CA* mutation, negative PTEN status, positive HER 2 status, or high IGF-1R.

^c^Covariates included age (≥65 versus <65 years), grade (grade 3 versus grades 1 to 2), tumor size (T3 to T4 versus T1 to T2), HER2 status (positive versus negative), and progesterone receptor status (positive versus negative).

### PIK3CA mutations have no prognostic effect in patients randomized to the control arm

In patients who did not receive tamoxifen, we did not observe an association between either *PIK3CA* mutation status, HER2, IGF-1R expression, or PTEN status and breast cancer prognosis (Table 4).

**Table 4:** Multivariate hazard ratios according to PI3K and/or MAPK molecular alterations in control patients

	***N *** **(events)**	**Hazard ratio**^**a**^	**95% confidence interval**	***P *** **value**
*PIK3CA* exon 9 mutant vs *PIK3CA* wt	111 (28)	0.49	0.11-2.25	0.36
*PIK3CA* exon 20 mutant vs *PIK3CA* wt	111 (28)	0.72	0.24-2.19	0.56
*PIK3CA* mutant (exon 9 or exon 20) vs *PIK3CA* wt	111 (28)	0.62	0.25-1.59	0.32
IGF-1R (3 versus 0–2)	96 (27)	1.81	0.67-4.87	0.24
PTEN positive versus negative	94 (25)	0.97	0.29-3.26	0.96
HER2 positive versus negative	121 (33)	0.60	0.13-2.76	0.51

^a^For each molecular alteration, Cox proportional hazard models were performed with co-variables: lymph node status, age, tumor size, grade, and progesterone receptor status. Mt, mutant; wt, wild type.

## Discussion

Our results indicate that *PIK3CA* mutations are unlikely to have important clinical validity to predict adjuvant tamoxifen resistance in postmenopausal breast cancer patients. In addition, we have shown that the presence of a molecular alteration in the PI3K and/or MAPK pathway is not always associated with high expression of downstream-activated proteins.

The observed frequency of *PIK3CA* mutations in the current study was in line with those reported in the literature [8,14,17]. Similar to others [16,31], we observed an association with low tumor grade and negative HER2 status. Although others did not observe a significant association between PIK3CA mutations and tumor grade [6,8,32], this discordance could be explained by the relatively small number of patients in these studies. In the studies from Buttitta [6] and Barbareschi [8], patients with lobular breast cancer had more often *PIK3CA* exon 9 mutated tumors compared with nonlobular breast cancer. In our study, we did not observe such enrichment for *PIK3CA* exon 9 mutations in lobular breast cancer. An important difference between these studies and our study population is that we selectively analyzed ERα-positive postmenopausal breast cancer patients, that are predominantly of low tumor grade. In the other studies [6,8], patients were younger, and the group of patients with nonlobular breast cancer included hormone receptor-negative patients, who are more often high tumor grade, and are less often *PIK3CA*-mutated [31]. This might explain why we observed a higher frequency of *PIK3CA* exon 9 mutations in nonlobular breast cancer compared with the studies from Buttita [6] and Barbareschi [8]. Similar to the results of *PIK3CA* mutation analysis in almost 2,000 patients who participated in the TEAM trial (treated with adjuvant tamoxifen and/or exemestane) [33], we observed a positive association between *PIK3CA* kinase domain mutations and PgR status. Altogether, our data indicate that in ERα-positive postmenopausal breast cancer patients, *PIK3CA* mutations are not enriched in lobular breast cancer, but are associated with favorable prognostic factors like low grade and positive PgR status.

In our study, we did not observe an association between *PIK3CA* mutations and activation of downstream proteins like mTOR and p70S6K. Although *in vitro* data have shown that *PIK3CA* mutations result in activation of the PI3K pathway [3], several studies have now shown that this is not necessarily the case in the clinical setting [14,34]. Perez *et al.*[14] did not find an association between *PIK3CA* mutations and p-AKT levels, whereas Loi *et al.*[34] observed relatively moderate activation of the PI3K pathway in tumors with a *PIK3CA* exon 20 mutation-associated gene signature. In addition, in a large series of primary breast cancers analyzed with exome sequencing and reverse-phase protein arrays, PI3K pathway activation was not elevated in *PIK3CA*-mutated luminal A cancers [35]. One of the explanations for this could be an AKT-independent downstream signaling in these *PIK3CA* mutated tumors [36]. Alternatively, relatively moderate pathway activation could be the result of a feedback mechanism leading to downregulation secondary to pathway activation. Previously it was shown that a negative-feedback loop between mTOR/p70S6K and the IRS protein results in a reduction of the IRS protein in response to activation of mTOR/p70S6K, with subsequent inhibition of the PI3K pathway [37].

In contrast, IGF-1R protein expression was significantly associated with p-p70S6K. Elevated IGF-1R signaling has been shown to result in activation of the PI3K and MAPK pathways *in vitro*[38]. Surprisingly, in tumors that scored negative for PTEN, we observed relatively low expression of downstream-activated proteins in the PI3K pathway. Although the robustness of PTEN antibodies has been a matter of debate, the reliability of the PTEN antibody we used was previously shown [39]. In addition, we showed a significant association between PTEN immunoscoring and mRNA expression. Similar to our results, Perez *et al*. [14] observed relatively low expression of AKT in patients whose tumor was negative for PTEN. The underlying mechanism for this unexpected observation remains unclear. An explanation could be that activation of the PI3K pathway in tumors that lack PTEN is relatively low compared with tumors that exhibit PI3K pathway activation from other causes.

In patients randomized to the control arm, we did not observe an association between *PIK3CA* mutations, or any of the other tested molecular aberrations, and breast cancer prognosis, when corrected for known prognostic factors, such as the PgR status and histologic tumor grade. The relatively low number of HER2-positive breast cancer patients in this series may explain the absence of a significant association between HER2 overexpression and breast cancer prognosis. It is well known that the incidence of HER2 overexpression in the Netherlands is lower than that observed in other countries [40]. With respect to the association of IGF-1R with breast cancer outcome, discordant results have been published [41-45]. These conflicting results may be explained by heterogeneous patient populations (like difference in histologic subtype and treatment) as well as differences in antibodies used.

The association between *PIK3CA* mutation and breast cancer prognosis has been controversial [31]. Previously, a *PIK3CA* exon 20 gene signature was associated with favorable outcome in both tamoxifen-treated patients and in patients who did not receive adjuvant systemic treatment [34]. *PIK3CA* mutation status, as defined with sequencing, did not have prognostic value in this study. Several other studies have suggested a favorable prognosis in patients harboring *PIK3CA* mutations [8,14]. A preliminary analysis of *PIK3CA* mutations in patients participating in the TEAM trial did not find a significant association with outcome [33]. In a recent meta-analysis showing a favorable clinical outcome in ERα-positive postmenopausal breast cancer patients with *PIK3CA* kinase domain mutations, a potential favorable response to endocrine treatment was suggested as one of the explanations [46]. Our results indicate that this is not likely to be true. A potential bias of these cohort studies is that the prognostic value of *PIK3CA* mutations is analyzed in breast cancer patients who all received adjuvant endocrine therapy. The prognosis of patients with *PIK3CA* mutations who have been treated with endocrine therapy is determined by both tumor biology and treatment effect, where the latter can range from substantial to no treatment effect. Hence its tumor biologic prognostic value cannot be deduced from such a study design.

The observed absence of a significant interaction between *PIK3CA* hotspot mutations and tamoxifen-treatment benefit in our study does not rule out a potential reduced effect of tamoxifen in patients carrying these hotspot mutations. Moreover, we cannot exclude that the benefit of tamoxifen might be reduced in patients whose tumors express a rare mutation in the *PIK3CA* gene or other genes in the PI3K/AKT/mTOR pathway (like *AKT1* mutations). Considering their relatively low prevalence [35], we did not determine these mutations, because the power of this study to demonstrate an interaction between these mutations, analyzed as single markers, and tamoxifen treatment is low. For similar reasons, we cannot exclude a potential reduced benefit from tamoxifen in patients whose tumors exhibit one of the canonic pathway drivers, like loss of PTEN and overexpression of HER2 and/or IGF-1R. Nevertheless, when a composed variable was tested, indicating the presence of any of these molecular alterations, again no significant interaction was found. We do not expect that the addition of other (rare) mutations in these analyses would substantially change these results.

In contrast, analysis of p-p70S6K identified a similar amount of patients and showed a highly significant test for interaction [29]. In *PIK3CA*-mutated tumors that do express p-p70S6K, a reduced effect is likely, but cannot be demonstrated because of lack of power. A meta-analysis of these markers on tumor material available from randomized clinical trials of adjuvant tamoxifen versus nil (for example, NSABP B-14 trial [47], NATO and CRC adjuvant breast trials [48,49], and the Stockholm trial [50]) might generate more definitive answers regarding these biomarkers.

Considering our observations in ERα-positive early breast cancer patients, it is not likely that *PIK3CA* hotspot mutation status by itself would be a suitable companion diagnostic predicting benefit from the putative use of PI3K/AKT/mTOR inhibitors in the adjuvant setting. Phase I data have shown a higher response rate for patients with gynecologic malignancies carrying a *PIK3CA* mutation treated with PI3K/AKT/mTOR inhibitors compared with patients with wild-type tumors [51]. However, whether this is also true for ERα-positive breast cancer must be defined in prospective randomized clinical trials, both in the metastatic and adjuvant settings, with these novel targeted agents. Our data indicate that the selection of patients for these trials should not be restricted to *PIK3CA* hotspot mutation carriers only.

## Conclusion

Novel targeted agents inhibiting the PI3K/AKT/mTOR pathway have a promising role in the treatment of patients with hormone receptor-positive breast cancer resistant to anti-estrogens as single agent. Because *PIK3CA* hotspot mutations frequently occur and are known to activate the PI3K/AKT/mTOR pathway, these mutations are generally considered a potential predictive biomarker. Our observations indicate that *PIK3CA* hotspot mutations have limited potential to predict intrinsic tamoxifen resistance in the adjuvant treatment of ERα-positive, postmenopausal breast cancer patients. Furthermore, no clear association between these mutations and activation of downstream proteins in the PI3K/AKT/mTOR pathway has been found in these patients. For identification of companion diagnostics, the focus should switch to the analysis of activated proteins downstream in the PI3K/AKT/mTOR pathway, which are associated with adjuvant tamoxifen resistance [29].

## Appendix 1

From paraffin-embedded tissue blocks, 10-μm-thick sections were cut and attached to microscope slides. In total, 10 slides per tumor were used for DNA isolation. Slides were deparaffinized in xylene, rehydrated, and stained with hematoxylin. The slides were incubated with sodium thiocyanate overnight. Exact tumor location was circled by a pathologist on an HE-stained slide, which was used as a template. After adding a drop of tissue lysis buffer, tumor tissue was scraped from the slides, added to a 1.5-μl micro centrifuge tube containing a 200-μl mix of tissue lysis buffer/proteinase K. This tube was incubated in a thermomixer at 55°C for 48 hours. An additional 27 μl proteinase K (2 mg/μl) was added after 24 and 36 hours. After 48 hours, the tube was incubated at 80°C for 10 minutes to inactivate proteinase K. After centrifuging, the supernatant was pipetted into a new tube. DNA was purified by using a QIAquick PCR purification kit.

## Abbreviations

CI: Confidence interval; ER: estrogen receptor; ERK: extracellular signal-regulated kinase; FFPE: formalin-fixed paraffin embedded; HER2: human epidermal growth factor receptor 2; HR: hazard ratio; IGF-1R: insulin-like growth factor 1 receptor; IHC: immunohistochemistry; MAPK: mitogen-activated protein kinase; mTOR: mammalian target of rapamycin; p: phosphorylated; PgR: progesterone receptor; PI3K: phosphatidylinositol-3-kinase; TMA: tissue microarray.

## Competing interests

The authors declare that they have no competing interests.

## Authors’ contributions

KB, SL, and AV were responsible for the concept and design of the study. TS, MO, RK, JV, PvD, JW, and JM contributed substantially to acquisition of the data. KB, AV, TS, EB, SL, JW, and PvD contributed to the analysis and interpretation of the data. KB, with supervision of SL, drafted the manuscript. All authors critically revised the manuscript for important intellectual content and approved the final version.

## Supplementary Material

Additional file 1: Table S1 Distribution of clinicopathologic variables between patients with sufficient tumor material for biomarker analysis and the total group of patients who entered the study. **Table S2**. Antibodies used for immunohistochemical assays. **Table S3**. Interobserver variability. **Table S4**. *PIK3CA* primers for MassARRAY. **Table S5**. Specifications of REMARK recommendations. **Table S6**. Frequencies of specific PIK3CA mutations in exon 9 **(A)**, exon 20 **(B)**, and the distribution of and IGF-1R protein expression intensity **(C)**. **Table S7**. Associations between PI3K/AKT/mTOR molecular alterations (columns) and downstream activated proteins (rows). Depicted are the *P* values for linear by linear tests. **Table S8**. Patient characteristics by treatment arm and *PIK3CA* mutation status. **Table S9**. Multivariate Cox proportional hazard model of recurrence-free interval (RFI) including PIK3CA mutation status and interaction with tamoxifen treatment. **Figure S1**. Membranous IGF-1R protein expression according to immunohistochemical staining of TMA cores from primary breast cancers compared with mRNA levels that were available from hybridization to a 44-K oligoarray (Agilent Technologies). In total, 40 cases of 69 patients were evaluable for IHC. In total, six IGF-1R probes were available, showing all similar results. The figure shows the data for the first IGF-1R probe (A_23_P205986). Linear-by-linear test was performed by using IGF-1R mRNA levels split by quartiles. **Figure S2**. Cytoplasmic PTEN protein expression according to immunohistochemical staining of TMA cores from primary breast cancers compared with mRNA levels that were available from hybridization to a 44K oligoarray (Agilent Technologies). In total, 36 cases of 69 patients were evaluable for IHC. In total, three PTEN probes were available, showing all similar results. The figure shows the data for the first PTEN probe (A_23_P98085). **Figure S3.** Data flow. Click here for file
