# Supplementary material for: PIK3CA mutations, phosphatase and tensin homolog, human epidermal growth factor receptor 2, and insulin-like growth factor 1 receptor and adjuvant tamoxifen resistance in postmenopausal breast cancer patients
Source: Breast Cancer Res. 2014 Jan 27;16(1):R13. doi: 10.1186/bcr3606 (PMC3978618; doi:10.1186/bcr3606)

Table S1 Distribution of clinic-pathological variables between patients with sufficient tumor material for biomarker analysis and the total group of patients who entered the study

		patients with sufficient tumor material	total study population
		N (%)	*N* (%)
	total	739 (100)	1662 (100)
**age**	<65	378 (51)	869 (52)
	≥ 65	361 (49)	793 (48)
**lymph node status**	negative	393 53)	901 (54)
	positive	346 (47)	761 (46)
**T stage**	T 1-2	659 (89)	1482 (89)
	T 3-4	80 (11)	180 (11)
**ERα status**[(1)]	positive	468 (77)	1014 (77)
	negative	142 (23)	311 (23)
**PgR status**[(1)]	positive	224 (57)	513 (60)
	negative	168 (43)	346 (40)

[(1)]As defined with ligand binding assay. After revision of recollected tumors, a total of 563 were ERα positive as assessed with immunohistochemistry

Table S2 Antibodies used for immunohistochemical assays

Protein name	Clone	Company	Art. No.
PTEN	138G6	Cell signaling	9559
IGF-1R	G11	Roche / Ventana	790-4346

Table S3: inter-observer variability

antibody	scoring system	comparable cores from N patients	weighted Kappa	cutoff used for binary score	Kappa for binary score
PTEN	cytoplasmic intensity	126	0.73 (0.64-0.81)	(1-3) versus 0	0.75
IGF-1R	membranous intensity	107	0.71 (0.61-0.79)	(0-2) versus 3	0.82

Table S4 *PIK3CA* primers for MassARRAY

Assay	*PIK3CA* mutation	position	wild type nucleotide	mutant nucleotide	2nd-PCR primer	1st-PCR primer	extension primer (UEP)	UEP MASS	UEP direction
1	exon 9 E542K	1624	G	A	ACGTTGGATGGCAATTTCTACACGAGATCC	ACGTTGGATGTAGCACTTACCTGTGACTCC	CACGAGATCCTCTCTCT	5081.3	F
2	exon 20 H1047L/R	3140	A	G/T	ACGTTGGATGTCCATTTTTGTTGTCCAGCC	ACGTTGGATGAACTGAGCAAGAGGCTTTGG	TTGTCCAGCCACCATGA	5130.4	R
3	exon 9 E545K	1633	G	A	ACGTTGGATGTAGCACTTACCTGTGACTCC	ACGTTGGATGGCAATTTCTACACGAGATCC	AGAAAATCTTTCTCCTGCT	5737.8	R

Table S5. Specifications of REMARK recommendations

**Introduction**	
Marker	Molecular alterations in PI3K/AKT/mTOR pathway : Hotspot mutations in *PIK3CA*, HER2 over-expression/amplification, IGF-1R over-expression and loss of PTEN
Objectives	To evaluate the predictive and prognostic capacity of the different molecular alterations in PI3K/AKT/mTOR pathway in postmenopausal breast cancer randomized between adjuvant tamoxifen versus control.
Hypothesis	The presence of these molecular alterations have been shown to result in *in vitro* activation of the PI3K/AKT/mTOR pathway, which results in endocrine resistance. We hypothesize that the presence of a molecular alteration in the PI3K/AKT/mTOR pathway is associated with clinical tamoxifen resistance.

**Methods (1)**	**Patients**
Characteristics	From 1982 until 1994 a randomized clinical trial was conducted in the Netherlands, studying the benefit of adjuvant tamoxifen (IKA-trial) in postmenopausal breast cancer patients.
Inclusion criteria	In the original study, 1662 breast cancer patients were included who were were post-menopausal, less than 76 years of age and had a $T_{1–4}$, $N_{0–3,}$ M_0 breast tumor. We have traced tissue blocks of participating patients and recollected sufficient tumor material of 739 patients, who did not differ in prognostic factors from the total group (Table S5). After revision of estrogen receptor α (ERα) status as assessed with immunohistochemistry (IHC), a total of 563 ERα positive (≥10%) tumors were used for subsequent analysis.
Exclusion criteria	Mastitis or palpable supra- or infraclavicular lymph nodes
Treatment	Patients were randomized in a 2:1 distribution between 1 year tamoxifen (30 mg per day) versus no adjuvant therapy. After 1 year a second randomization was performed to receive another 2 years of tamoxifen or to stop further treatment. From 1989, based on two interim analyses showing a significant improvement in recurrence free-free survival in lymph node positive patients, these node positive patients were all allocated to the tamoxifen treatment arm (ie skipped the first randomization).

**Methods (2)**	**Specimen characteristics**
Material used	Formalin-fixed paraffin-embedded (FFPE) breast tumor tissue of the primary tumor. DNA was isolated from FFPE material.
Preservation/storage	Formalin fixation and paraffin embedding. Storage at room temperature. Tumor DNA was stored at 4ºC.

Methods (3)	Assay methods
Assay	Genotyping for PIK3CA exon 9 (E542K and E545K) and exon 20 mutations (H1047L and H1047R) was performed on genomic DNA using Sequenom mass spectrometry–based genotyping technology. HER2 status was assessed with a standard immunohistochemistry protocol and considered positive when membranous staining was DAKO score 3. In case of a DAKO score 2, Silver *in situ* Hybridization was performed using ultraView SISH Detection Kit (Ventana ®) according to the manufacturer's instructions and amplified cases were considered as HER2 positive. Immunohistochemistry for IGF-1R (Ventana anti-IGF-1R rabbit monoclonal antibody) and PTEN (Cell Signaling # 9559) was performed using the Ventana Benchmark® Ultra system.
Protocol	PCR primers and extension primers for the various PIK3CA mutations are listed in Table S8. Immunohistochemical stainings were performed using a standardized protocol on the Ventana Benchmark® Ultra system. Staining protocols can be downloaded from our website: http://research.nki.nl/linnlab/index.htm
Control experiments	The specificity of the PTEN and IGF-1R antibodies was tested on a previously described series of metastatic breast cancer patients [21] for which we had FFPE material embedded in a TMA as well as Agilent 44K mRNA expression data. Results are depicted in Figure S1-2. For genotyping for PIK3CA mutation status control experiments were performed using PCR and capillary sequencing. For exon 9, a total of 349 samples could be compared between the two techniques (Sequenom mass spectrometry–based genotyping technology versus PCR and capillary sequencing) resulting in 91 % concordant results. In 8% of the cases a mutation was found with Sequenom mass spectrometry–based genotyping which was not detected with PCR and capillary sequencing, while in 1% of the cases a a mutation was found with PCR and capillary sequencing which was not detected with Sequenom mass spectrometry–based genotyping. For exon 20, a total of 408 samples could be compared between the two techniques (Sequenom mass spectrometry–based genotyping technology versus PCR and capillary sequencing) resulting in 96 % concordant results. In 3% of the cases a mutation was found with Sequenom mass spectrometry–based genotyping which was not detected with PCR and capillary sequencing, while in 1% of the cases a mutation was found with PCR and capillary sequencing which was not detected with Sequenom mass spectrometry–based genotyping.
Reproducibility	For each immunohistochemical staining, one of the TMAs was quantified independently in a blinded manner by a second observer to calculate inter-observer variability. The inter-observer variability analyzed using the (weighted) Cohen's kappa coefficient is depicted in Table S3
Quantification	Quantification of immunohistochemical staining was performed as described in the method section for immunohistochemistry.
Blinding	Scoring of the immunohistochemical stainings was done without knowledge regarding both the recurrence-free-interval survival as well as the treatment arm at the time of scoring.

Methods (4)	Study design I
Case selection	A randomized controlled trial. The translational study presented her was performed retrospectively. The median duration of follow-up for patients without a recurrence event was 7.8 years. Patient records were re-evaluated for recurrence until 2000.
Clinical endpoints	The improvement of recurrence free interval (RFI) with tamoxifen versus nil was assessed according to the different levels of the tested drivers and downstream activated proteins as specified below. RFI included local, regional, distant recurrences and breast cancer-specific death, but not contra-lateral breast cancer, as the primary event.
Variables examined or considered	Multivariate Cox models included age (≥ 65 versus < 65), grade (grade 3 versus grade 1-2), tumor size (T3-4 versus T1-T2), HER2 status (positive versus negative) and progesterone status (positive versus negative) as covariates.
Rational for sample size	The sample size of the translational study is based on the amount of available tumor blocks containing invasive, ERα positive tumor cells, that could be recollected and a power calculation based on events in this group assuring that meaningful results could be deduced.

Methods (5)	Statistical analysis
Statistical methods and variable selection procedure	Recurrence free interval was defined as the time from the date of first randomization until the occurrence of a local, regional or distant recurrence or breast cancer specific death. A secondary contra-lateral breast tumor was not considered as an event and these patients were censored at the date of this occurrence. The association of *PIK3CA* mutations, PTEN, HER2 and IGF-1R protein expression with expression of downstream activated proteins in the PI3K/AKT/mTORpathway (like p-AKT, p-mTOR and p-p70S6K) was evaluated using linear by linear tests. Survival curves were constructed using the Kaplan-Meier method. All survival analyses were stratified for nodal status.All p-values are based on a two-sided test. All calculations were made with Statistical Package for the Social Sciences (SPSS) 15.0 Inc., IL, USA.
Missing data	Cases with a missing value for one of the variables were excluded from the multivariate analysis, with the exception of missing HER2 and PgR data for which a separate level was created
Marker handling in analysis	In our primary analysis we tested the clinical validity of these molecular alterations as single markers, analyzed as binary factor. Covariate adjusted Cox proportional hazard regression analyses were performed including an interaction variable. The following molecular alterations were tested: PIK3CA mutation status (exon 9 mutant versus exon 9 wild type and exon 20 mutant versus exon 20 wild type imputed as separate factors in one model), HER2 (positive versus negative), PTEN (negative versus positive) and IGF-1R (score 3 versus score 0-2). In addition, we tested the interaction with tamoxifen for a composed variable defined as any of these molecular alterations present versus no molecular alteration.<br>Covariates included age (≥ 65 versus < 65), grade (grade 3 versus grade 1-2), tumor size (T3-4 versus T1-T2), HER2 status (positive versus negative), and PgR status (positive versus negative). Due to multiple co-primary endpoints, the level of significance was set at 0.01.<br>In addition we tested the prognostic effects of these molecular alterations in control patients using covariate adjusted Cox proportional hazard regression analyses.

Results (1)	Data
Flow of patients	See Figure S3 for description of patients excluded for this translational study. See Table S1 for characteristics of total study patients versus the 739 patients with sufficient tumor material included in TMA .
Characteristics	See Table 1 and Supplementary Table S8.

Results (2)	Analysis and presentation
Relation to standard prognostic variables	See Table 1.
Univariate analysis	Data not shown
Multivariate analysis:	See Tables 2 and 3 and Supplementary Table S9. Estimated effects with CIs for marker and all other variables in the model.

Discussion	
Interpretation, limitations and implication	See discussion section

Table S6: Frequencies of specific PIK3CA mutations in exon 9 (A), exon 20 (B) and the distribution of and IGF-1R protein expression intensity (C)

A.

**PIK3CA exon 9**	**N**	**%**
wild type	412	84
exon9G1633A (E545K)	45	9
exon9G1624A (E542K)	31	6
Total	488	100

B.

**PIK3CA exon 20**	**N**	**%**
wild type	402	82
exon20A3140G (H1047R)	79	16
exon20A3140T (H1047L)	10	2
Total	491	100

C.

**IGF-1R membranous intensity**	**N**	**%**
0	49	11
1	120	26
2	231	53
3	39	9
Total	439	100

Table S7: Associations between PI3K/AKT/mTOR molecular alterations (colums) and downstream activated proteins (rows). Depicted are the p-values for linear by linear tests.

		***PIK3CA* exon 20**	***PIK3CA* exon 9**	***PIK3CA* exon 9 or exon 20**	**HER2 status**	**PTEN**	**IGF-1R**	**Any PI3K pathway driver**
	**variable levels**	mutant vs wt	mutant vs wt	mutant vs wt	positive versus negative	negative versus positive	0-3	present[(1)] versus absent
**p-AKT(Ser473)**	0-3	0.64	0.80	0.47	0.52	<0.0001	<0.0001	0.14
**p-AKT(Thr308)**	0-3	0.14	0.03	0.02	0.91	0.02	0.48	0.15
**p-mTOR**	0-100	0.34	0.65	0.26	0.21	0.002	0.28	0.12
**p-ERK1/2**	0-100	0.04	0.06	0.007	0.72	0.001	0.71	0.40
**p-p70S6K**	0-3	0.36	0.34	0.92	0.23	<0.0001	<0.0001	0.28

[(1)]defined as either *PIK3CA* mutant present, HER2 over-expressed, PTEN negative or IGF-1R intensity score 3. wt=wild type

(red) marker of PI3K pathway activation positively associated with presence of PI3K pathway driver

(green) marker of PI3K pathway activation negatively associated with presence of PI3K pathway driver

Table S8 Patient characteristics by treatment arm and *PIK3CA* mutation status

		PIK3CA								
		wild type			exon 9 mutant			exon 20 mutant		
		control	tamoxifen	p-value(1)	control	tamoxifen	p-value(1)	control	tamoxifen	p-value(1)
		N (%)	N (%)		N (%)	N (%)		N (%)	N (%)	
**age**	<65	42 (53)	127 (50)	0.80	8 (47)	22 (37)	0.58	13 (57)	26 (39)	0.22
	≥65	38 (48)	125 (50)		9 (53)	37 (63)		10 (44)	40 (61)	
**lymph node status**	negative	64 (80)	114 (45)	<0.0001	13 (77)	26 (44)	0.03	20 (87)	35 (53)	0.005
	positive	16 (20)	138 (55)		4 (24)	33 (56)		3 (13)	31 (47)	
**T stage**	T1-2	73 (91)	224 (89)	0.68	16 (94)	53 (90)	1.00	22 (96)	57 (86)	0.44
	T3-4	7 (9)	28 (11)		1 (6)	6 (10)		1 (4)	9 (14)	
**grade**	grade 1-2	43 (54)	157 (62)	0.19	16 (94)	43 (73)	0.10	18 (78)	47 (71)	0.59
	grade 3	37 (46)	95 (38)		1 (6)	16 (27)		5 (22)	19 (29)	
**progesterone receptor**	negative	40 (50)	128 (51)	0.90	7 (41)	28 (47)	0.78	10 (43)	24 (36)	0.46
	positive	40 (50)	120 (48)		9 (53)	29 (49)		12 (52)	42 (64)	
	missing	0 (0)	4 (2)		1 (6)	2 (3)		1 (4)	0 (0)	
**HER2**	negative	70 (88)	214 (85)	0.38	15 (88)	56 (95)	0.22	20 (87)	60 (91)	0.33
	positive	5 (6)	26 (10)		1 (6)	0 (0)		0 (0)	5 (8)	
	missing	5 (6)	12 (5)		1 (6)	3 (5)		3 (13)	1 (2)	

(1)Fisher exact tests, only cases without missing values were analyzed

Table S9 Multivariate Cox proportional hazard model of recurrence free interval (RFI) including PIK3CA mutation status and interaction with tamoxifen treatment

		[redacted]		
**Variable**		**Hazard Ratio**[1]	**95% CI**	**p-value**
**Age**				
< 65	216(56)	ref		
≥ 65	241 (51)	0.88	0.59-1.31	0.53
**p T-stage**				
T1 or T2	408 (87)	ref		
T3 or T4	49 (20)	1.56	0.93-2.60	0.09
**Histologic grade**				
I-II	297 (56)	ref		
III	160 (51)	1.60	1.05-2.44	0.03
**Progesterone receptor**				
negative	223 (47)	ref		
positive	234 (60)	1.21	0.81-1.80	0.36
**HER2 status**				
negative	422 (96)	ref		
positive	35 (11)	1.17	0.60-2.27	0.64
**PIK3CA exon 9**				
no mutation	386 (93)	ref	0.23-2.70	0.70
mutant[2]	71(14)	0.79		
**PIK3CA exon 20**				
no mutation	375 (87)	ref	0.28-2.46	0.74
mutant[2]	82 (20)	0.83		
**Treatment**				
PIK3CA wild type and control	74 (21)	ref	0.30-0.88	0.02
PIK3CA wild type and tamoxifen	234 (54)	0.51		
PIK3CA exon 9 mutant and control	16 (3)	ref		
PIK3CA exon 9 mutant and tamoxifen	55 (11)	0.82	0.22-3.04	0.77
PIK3CA exon 20 mutant and control	18 (4)	ref		
PIK3CA exon 20 mutant and tamoxifen	64 (16)	0.77	0.25-2.36	0.65
interaction PIK3CA (ex 9) status X treatment				0.51
interaction PIK3CA (ex 20) status X treatment				0.51

(1)stratified for nodal status ,
(2) 4 out of these had both exon 9 and exon 20 mutation
Analysis based on 457 cases with 107 events

Figure S1: Membranous IGF-1R protein expression according to immunohistochemical staining of TMA cores from primary breast cancers compared to mRNA levels that were available from hybridization to a 44K oligoarray (Agilent Technologies). In total 40 cases out of 69 patients were evaluable for IHC. In total 6 IGF-1R probes were available, showing all similar results. The figure shows the data for the first IGF-1R probe (A_23_P205986). Linear by linear test was performed using IGF-1R mRNA levels split by quartiles.

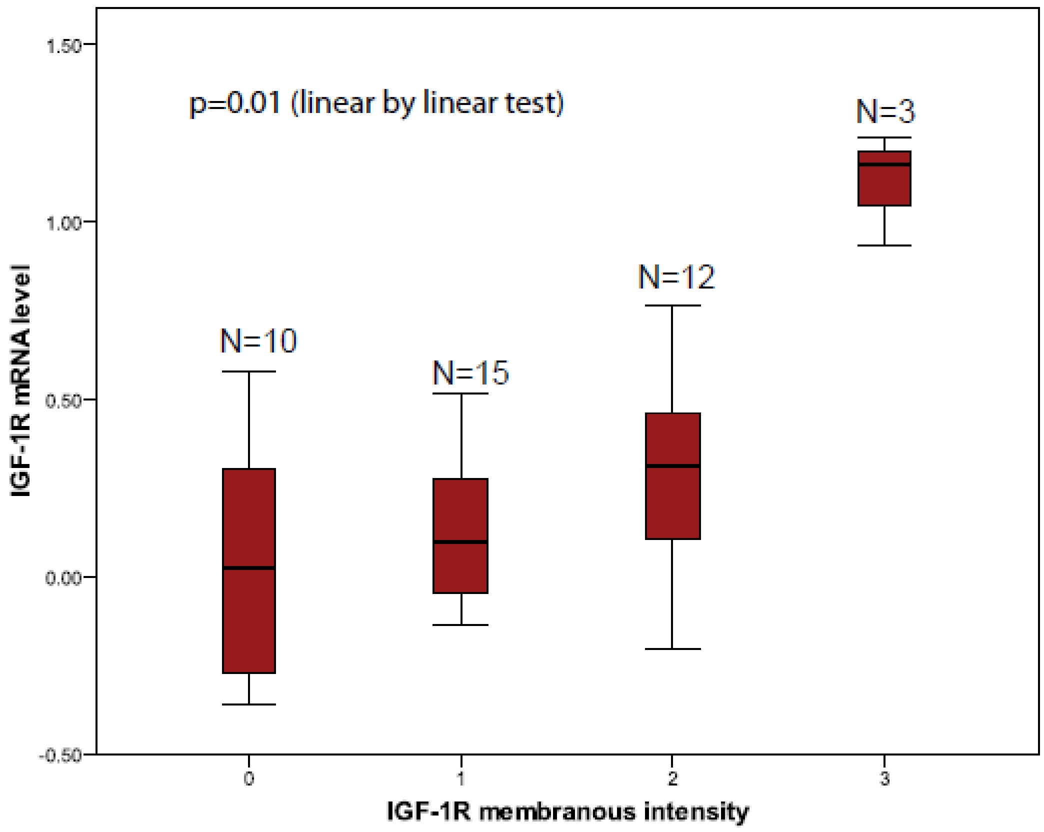

Figure S2: Cytoplasmic PTEN protein expression according to immunohistochemical staining of TMA cores from primary breast cancers compared to mRNA levels that were available from hybridization to a 44K oligoarray (Agilent Technologies). In total 36 cases out of 69 patients were evaluable for IHC. In total 3 PTEN probes were available, showing all similar results. The figure shows the data for the first PTEN probe (A_23_P98085)

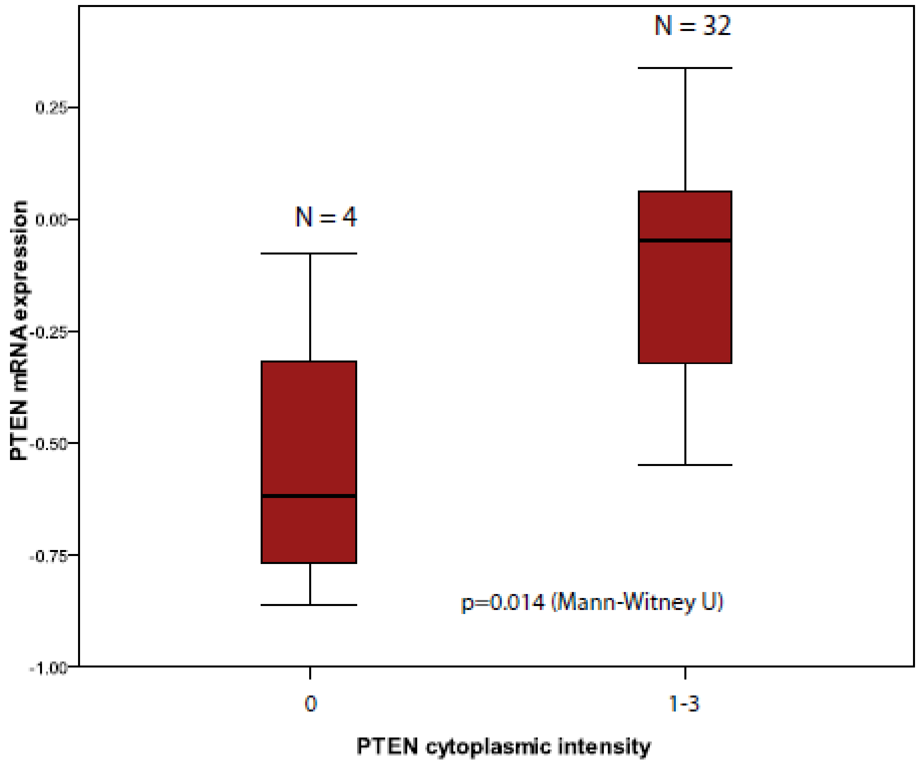

Figure S3 Data flow

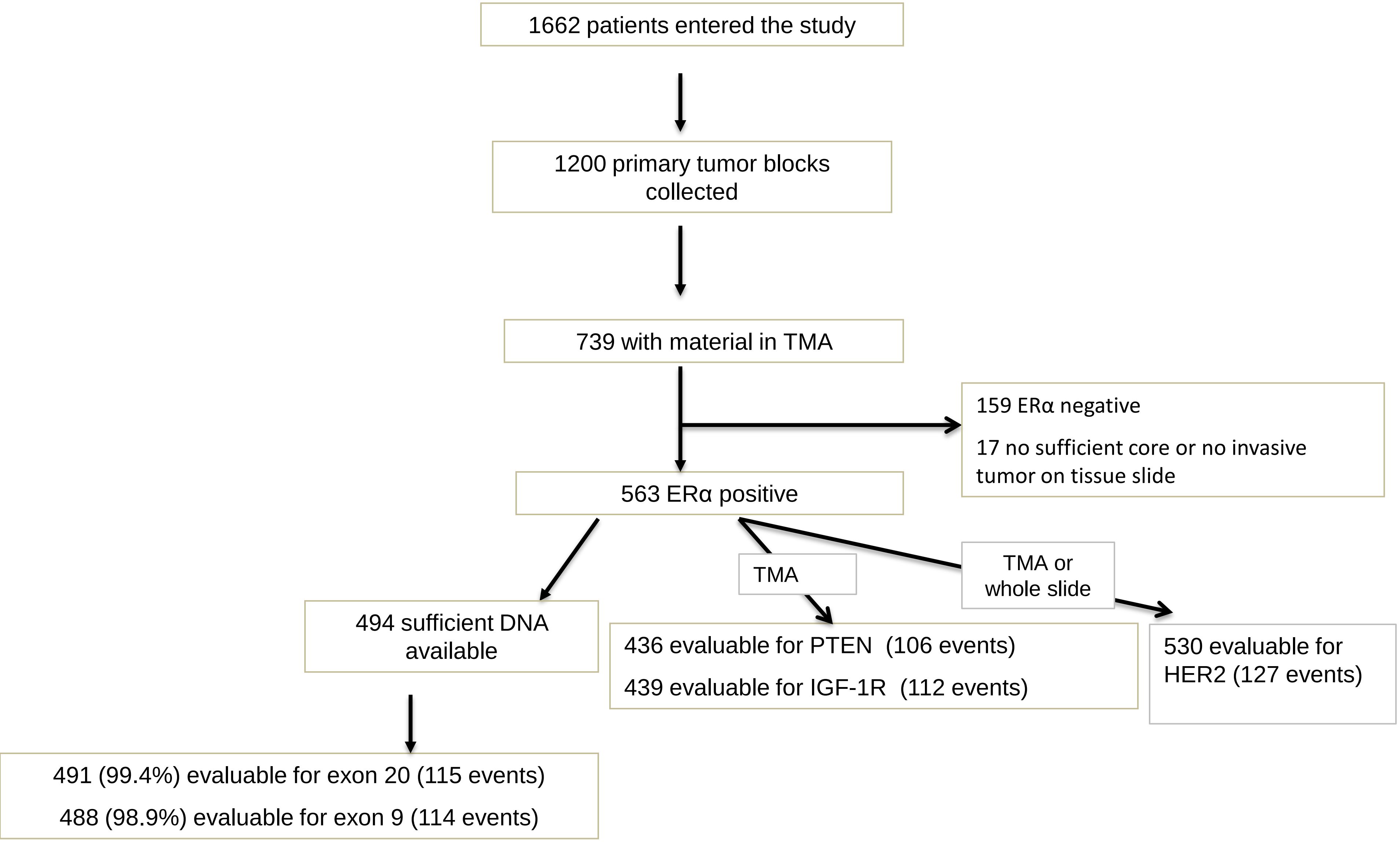

Supplement: Additional file 1: Table S1 — Distribution of clinicopathologic variables between patients with sufficient tumor material for biomarker analysis and the total group of patients who entered the study. Table S2. Antibodies used for immunohistochemical assays. Table S3. Interobserver variability. Table S4. PIK3CA primers for MassARRAY. Table S5. Specifications of REMARK recommendations. Table S6. Frequencies of specific PIK3CA mutations in exon 9 (A), exon 20 (B), and the distribution of and IGF-1R protein expression intensity (C). Table S7. Associations between PI3K/AKT/mTOR molecular alterations (columns) and downstream activated proteins (rows). Depicted are the P values for linear by linear tests. Table S8. Patient characteristics by treatment arm and PIK3CA mutation status. Table S9. Multivariate Cox proportional hazard model of recurrence-free interval (RFI) including PIK3CA mutation status and interaction with tamoxifen treatment. Figure S1. Membranous IGF-1R protein expression according to immunohistochemical staining of TMA cores from primary breast cancers compared with mRNA levels that were available from hybridization to a 44-K oligoarray (Agilent Technologies). In total, 40 cases of 69 patients were evaluable for IHC. In total, six IGF-1R probes were available, showing all similar results. The figure shows the data for the first IGF-1R probe (A_23_P205986). Linear-by-linear test was performed by using IGF-1R mRNA levels split by quartiles. Figure S2. Cytoplasmic PTEN protein expression according to immunohistochemical staining of TMA cores from primary breast cancers compared with mRNA levels that were available from hybridization to a 44K oligoarray (Agilent Technologies). In total, 36 cases of 69 patients were evaluable for IHC. In total, three PTEN probes were available, showing all similar results. The figure shows the data for the first PTEN probe (A_23_P98085). Figure S3. Data flow. [file bcr3606-S1.pdf]
